# Chaperonin Abundance Enhances Bacterial Fitness

**DOI:** 10.3389/fmolb.2021.669996

**Published:** 2021-07-26

**Authors:** C. M. Santosh Kumar, Kritika Chugh, Anirban Dutta, Vishnuvardhan Mahamkali, Tungadri Bose, Sharmila S. Mande, Shekhar C. Mande, Peter A. Lund

**Affiliations:** ^1^ School of Biosciences and Institute of Microbiology and Infection, University of Birmingham, Birmingham, United Kingdom; ^2^ Department of Biotechnology and Bioinformatics, University of Rajasthan, Jaipur, India; ^3^ TCS Research, Tata Consultancy Services Ltd., Pune, India; ^4^ Australian Institute for Bioengineering and Nanotechnology (AIBN), The University of Queensland, Brisbane, QLD, Australia; ^5^ Laboratory of Structural Biology, National Centre for Cell Science (NCCS), Pune, India

**Keywords:** metabolic flux, GroEL, evolution, proteomics, metabolism, competitive index

## Abstract

The ability of chaperonins to buffer mutations that affect protein folding pathways suggests that their abundance should be evolutionarily advantageous. Here, we investigate the effect of chaperonin overproduction on cellular fitness in *Escherichia coli*. We demonstrate that chaperonin abundance confers 1) an ability to tolerate higher temperatures, 2) improved cellular fitness, and 3) enhanced folding of metabolic enzymes, which is expected to lead to enhanced energy harvesting potential.

## Introduction

Chaperonins are found in nearly every organism across all domains of life, and are essential in all cases tested to date, although in some cases non-essential paralogues are found (Lund, 2009; Kumar, 2017). The GroE chaperonin system of *E. coli*, consisting of the 60 kDa GroEL and the 10 kDa GroES proteins assembled into ring complexes of 14 and seven sub-units, respectively, is encoded by the *groE* operon (Tilly and Georgopoulos, 1982; Bukau and Horwich, 1998; Balchin et al., 2016). This operon is expressed principally from two promoters, one utilized in the presence of housekeeping sigma factor *σ* ^70^, and the other, which is strongly induced due to the accumulation of unfolded proteins, in the presence of the alternative sigma factor, *σ* ^32^ (RpoH) (Kusukawa and Yura, 1988; Lund, 2001; Kumar et al., 2015; Schumann, 2017). As *σ* ^32^ levels respond to unfolded protein, this provides a feedback loop to maintain proteostasis (Kim et al., 2013). When cells are shifted to heat shock temperatures between 42 and 46°C, GroEL levels increase by 5–10 fold, reaching up to 12% of the entire cellular proteome (Martin et al., 1992). These increased levels interact more extensively with the proteome and are assumed to prevent misfolding or assist refolding of heat-stressed proteins (Martin et al., 1992; Llorca et al., 1998; Houry et al., 1999). Cells that cannot mount an unfolded protein response due to *rpoH* deletion are extremely temperature sensitive, and selection for pseudo-revertants of these strains at elevated temperatures yields up-promoter mutations in the *groE* promoter (Kusukawa and Yura, 1988). GroE is thus important even under normal growth conditions, and indeed GroEL and GroES are respectively the 20th and 21st most abundant proteins in *E. coli* (excluding ribosomal proteins), with sufficient protein being made under non-stressed conditions to produce approximately 2,800 complexes of GroEL and 5,700 complexes of GroES (Li et al., 2014). Other chaperones that are also abundant include the ribosome bound trigger factor (TF), which is the 19th, and the Hsp70 homologue, DnaK, which is the 27th most abundant. The high levels of all these chaperones indicates their key roles in cell growth. Although combined loss of TF and DnaK is deleterious to cells, *groEL* and *groES* are the only chaperone encoding genes in *E. coli* that are essential under all conditions (Fayet et al., 1989).

GroE (GroEL and GroES) assists the folding of 10–15% cellular proteins (Houry et al., 1999), many of which are essential (Kerner et al., 2005). GroE’s ability to fold “folding-compromised” proteins (Houry et al., 1999; Fares et al., 2002; Kerner et al., 2005; Tokuriki and Tawfik, 2009) is consistent with a “genetic capacitance” function. Many studies with different heterologous proteins have shown that GroE can enhance their folding (Tokuriki et al., 2008; Tokuriki and Tawfik, 2009; Wyganowski et al., 2013; Ishimoto et al., 2014; Durao et al., 2015). In addition, some deleterious mutations are retained in the genome upon overexpression of *groE*, probably due to chaperonin-buffered folding of polypeptides whose folding pathway has been perturbed (Van Dyk et al., 1989; Fares et al., 2002; Williams and Fares, 2010; Sabater-Munoz et al., 2015). However, since GroE is an active ATPase, its overproduction could be deleterious to the cell, owing to the depletion of cellular energy pools. Here, we have assessed the effect of GroE overproduction on the growth characteristics and thermal tolerance of *E. coli* and used proteomics and *in silico* flux balance analysis (FBA) to determine the likely impact of chaperonin overproduction on the metabolic advantage and consequent fitness of the organism.

## Materials and Methods

### Materials, Plasmids, Bacterial Strains and Growth Conditions

All chemicals were from Sigma, Inc. Bacterial growth media and media supplements were from HiMedia Laboratories, Inc., Mumbai, India. Phusion polymerase for colony PCR was purchased from New England Biolabs Inc., United States. GroE expression plasmids, pBAD-GSL and pTrc-GSL were generated by cloning GroE operon into NcoI and HindIII sites on plasmids pBAD24 (Guzman et al., 1995) and pTrc99A (Amann et al., 1988), respectively. The *groE* conditional mutant strain, *E. coli* LG6, was a kind gift from Arthur Horwich, Yale University, United States (Horwich et al., 1993). This strain produces GroE at levels similar to the wildtype at 30°C upon induction (Supplementary Figure 1). Oligonucleotide primers were purchased from Integrated DNA Technologies, Inc., Coralville, IA, United States.

### Construction and Validation of Strains Producing High and Low GroE Levels

**FIGURE 1:**
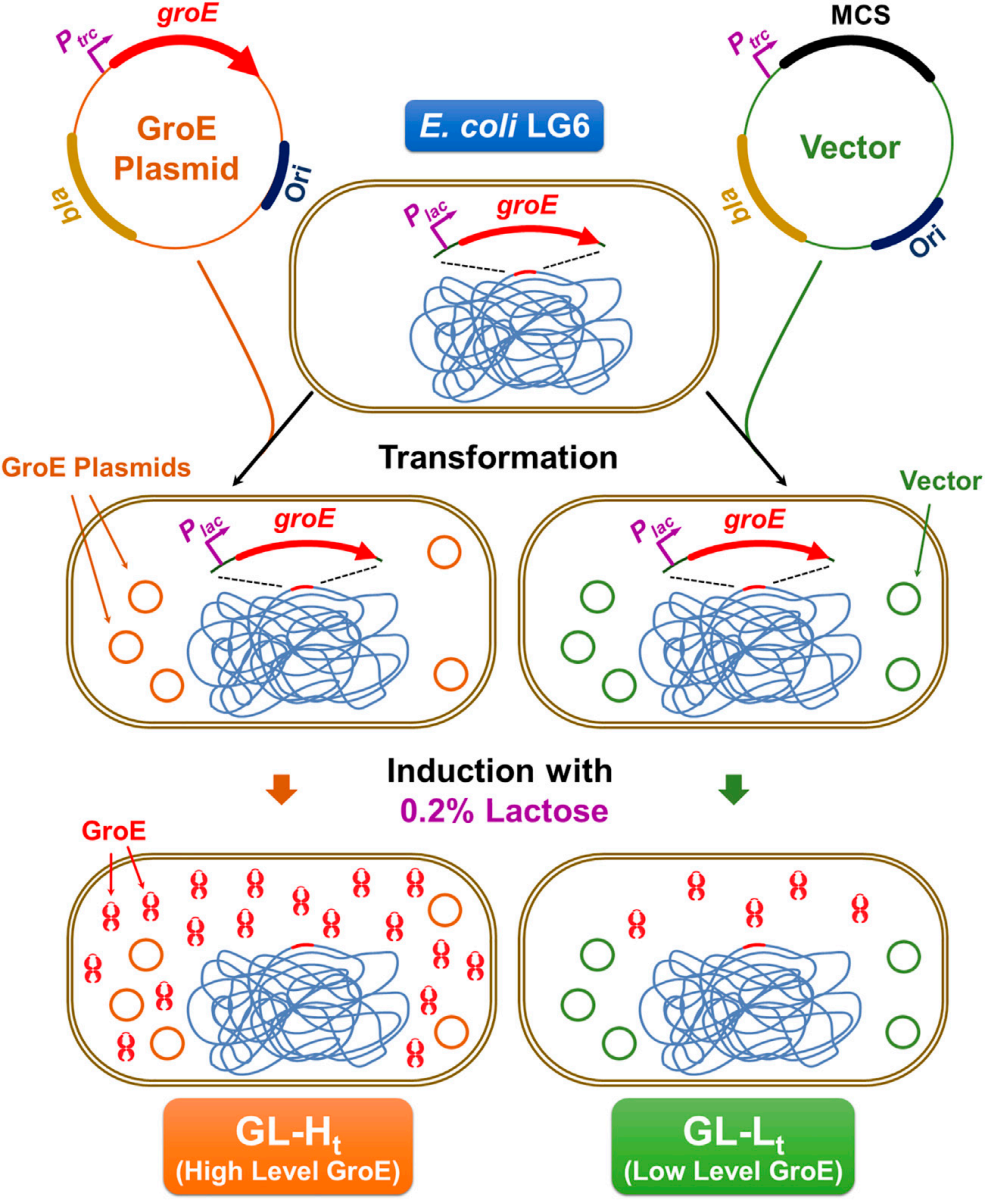
Construction of GL-L_t_ and GL-H_t_ Strains. In *E. coli* LG6, the *groE* operon is under the control of the inducible *P*_*trc*_ promoter. This strain was transformed with either a plasmid expressing *groE* under the control of lactose (GroE Plasmid), pTrc-GSL or its empty vector (Vector), pTrc99a. Upon culturing in the presence of lactose, GL-H_t_ produces elevated levels of GroEL and GroES, due to the induction of chromosomal and plasmid-borne *groE* operon, while GL-L_t_ will have lower production of GroES and GroEL due to the induction of only the chromosomal *groE* copy.

To enable control of GroE levels independently from the growth temperature, two strains that differentially express *groE* were generated from the *E. coli* strain LG6, in which the chromosomal *groE* promoter has been replaced with a *P*_*lac*_ promoter (Horwich et al., 1993). A high level GroE expression strain, GL-H_t_ (for GroEL High pTrc), was obtained by transforming LG6 with pTrc-GSL and a lower level GroE expression strain, GL-L_t_ (for GroEL Low pTrc) was obtained by transforming with the control plasmid pTrc99A (Amann et al., 1988). The scheme for the generation of these phenotypes is illustrated in Figure 1. To confirm the expression levels, these strains were cultured in the presence of 0.2% D-lactose to induce chromosome and plasmid borne *groE* operons, for 3 h at 30°C. The resulting cells were suspended in lysis buffer containing 50 mM HEPES:KOH pH 7.5 and 150 mM NaCl, 1 mM EDTA, and 1 mM PMSF, mixed with Lysing Matrix E and lysed by homogenization in FastPrep (M. P. Biomedicals, Irvine, CA, United States). Lysates were centrifuged at 13,000 rpm for 20 min to obtain soluble lysates. The soluble lysates were resolved on 12.5% SDS-PAGE and 12% Tricine gel followed by Coomassie Brilliant Blue staining to detect the levels of GroEL and GroES, respectively. In parallel, these lysates were probed with an anti-GroEL monoclonal antibody (1.10B) at 1:100 dilution and the blots were developed by BCIP/NBT-Purple Liquid Substrate System (Sigma Aldrich Inc., St. Louis, MO, United States). In addition to these strains, two strains that enable independent regulation of the chromosome and plasmid borne copies of *groE* operon were generated by transforming LG6 with pBAD-GSL and pBAD24 to result in high and low expression strains, GL-H_b_ and GL-L_b_, respectively. These strains were cultured in the presence of 0.2% lactose plus 0.2% arabinose to obtain the high and low expression levels (Supplementary Figure 2A).

### Temperature Sensitivity Assessment

The extent to which GroE overproduction enables temperature tolerance was assessed using a complementation assay (Kumar et al., 2009; Chilukoti et al., 2015). Actively growing cultures of GL-H_t_ and GL-L_t_ were normalized for OD_600_, serially diluted, and spotted onto eight LB agar plates supplemented with 0.2% D-lactose. The plates were incubated at 17, 20, 22, 25, 30, 37, 40, 42, 45, 46, and 48°C. Wild type MG1655 harboring pTrc-GSL or empty vector (pTrc99A), respectively, were included as controls.

### Competition and Estimation of Relative Fitness

GL-H_b_ and GL-L_b_ cells were subjected to competitive serial culturing as described previously (Zambrano et al., 1993; Vulic and Kolter, 2001; Smith, 2011). Briefly, equal number of cells from these two cultures were mixed and grown in fresh LB supplemented with 0.2% L-arabinose and 0.2% D-lactose. This mixed culture was grown to stationary phase at 30°C, recovered, labelled Passage-1 and used to generate the second passage (Figure 3A). Serial sub-culturing was repeated for a further 20 passages (∼700 generations). At each passage, a fraction of the cultures was serially diluted up to 10^−7^ dilution in LB broth and spread on LB agar plates supplemented with 0.2% D-lactose, which supports the growth of the cells derived from either strain. The resulting colonies at each passage, in the range of 23–28 colonies, were screened using colony PCR to identify whether colonies were derived from either GL-H_b_ or GL-L_b_ cells. Colony PCR with the PBADF (5′-CTG​TTT​CTC​CAT​ACC​CGT​T-3′) and PBADR (5′-CTC​ATC​CGC​CAA​AAC​AG-3′) primers, which bind upstream and downstream of the MCS on the parental vector pBAD24, results in the amplification of 2.1 and 0.3 kb fragments from the pBAD-GSL and pBAD24 vectors, harbored by the GL-H_b_ and GL-L_b_ cells, respectively. Relative competitive index (CI), a measure of relative fitness, was calculated for each phenotype as the ratio of the proportion of a particular cell type at the final and initial generations (Monk et al., 2008; Macho et al., 2010; van Opijnen and Camilli, 2013).

### Proteomic Analysis

Equal number of cells from exponentially growing cultures (OD_600_ = ∼0.6) of GL-H_b_ or GL-L_b_ strains were harvested, suspended in lysis buffer (50 mM HEPES:KOH pH: 7.5 and 150 mM NaCl, 1 mM EDTA, and 1 mM PMSF), lysed by sonication, and the soluble protein fractions were recovered by centrifugation at 12,000 rpm for 20 min 200 µg protein from the soluble fractions of each lysate were resolved through 2D PAGE following the standard protocols. Briefly, the lysates were resolved on the first dimension through a 7 cm Immobilized pH Gradient (IPG) strip of 3–10 pH range, followed by 10% SDS-PAGE on the second dimension. The separated proteins were stained with Coomassie brilliant blue and intensities of the stained protein spots were compared between the two gels using densitometry. This experiment was repeated three times to identify the spots that exhibited consistent differential enrichment between the strains. Differentially enriched spots between the two lysates were picked and identified by tandem mass-spectrometry in an LTQ Orbitrap Mass Spectrometer (Thermo Fisher Scientific Inc., Waltham, MA, United States). The differentially enriched proteins were identified using MASCOT (Hirosawa et al., 1993) search against UniProtKB/TrEMBL (UniProt, 2019) and RefSeq (O'Leary et al., 2016) databases. The spot identification was done in collaboration with the Centre for Cellular and Molecular Platforms, Bangalore, India.

### Flux Balance Analysis of the GL-H_b_ and GL-L_b_ Strains

*E. coli* genome-scale metabolic network iJO1366 (Blais et al., 2013) was used for performing the FBA simulations. The iJO1366 model was first simulated using a standard energy source (equivalent of a glucose-supplemented minimal media) to obtain the steady state fluxes through each of the reactions (Orth et al., 2011). The objective function of this FBA simulation was to maximize the biomass production, while using some “default constraints” (lower- and upper-bounds of fluxes through each reaction) derived from the literature (Blais et al., 2013). Following this preliminary assessment of the *E. coli* cell’s metabolic potential, two independent FBA simulations were performed, each of which corresponded to the enzyme expression/enrichment profiles of the GL-H_b_ and GL-L_b_ strains. During each of these simulations the reaction flux values were appropriately constrained, based on the results from the preliminary assessment and the corresponding enzyme expression/enrichment profiles (Supplementary File 2). Incorporating enzyme expression profiles into FBA simulations was performed with our software tool “TransFlux,” developed in-house, and housed at http://www.nccs.res.in/TrasFlux/index.jsp. Details of the parameters and the principles applied in FBA are presented in the Supplementary Material methods section.

## Results

### Construction of GroE Overproducing Strains

To investigate the effect of chaperonin overproduction on *E. coli*, we constructed two chaperonin producing strains, GL-H_t_ and GL-L_t_, which produce high and low levels of GroE (Figure 1). These strains were derived from strain *E. coli* LG6 (Horwich et al., 1993), in which the *P*_*groE*_ promoter is replaced by the *P*_*lac*_ promoter, by transforming with pTrc-GSL, which overexpress *groE* operon upon induction with lactose, or its parental plasmid pTrc99A. SDS-PAGE confirmed significant overproduction of GroEL (Supplementary Figure 1A) in GL-H_t_ compared to GL-L_t_. From Western blotting of the lysates, we estimate that GroEL levels are twenty-fold greater in GL-H_t_ than in GL-L_t_ (Supplementary Figure 1B). The expression levels of GroEL in GL-L_t_ were lower than the MG1655, where wildtype *P*_*groE*_ promoter drives the expression (Supplementary Figure 1) (Chapman et al., 2006). Further, GroES was significantly overproduced in GL-H_t_ compared to GL-L_t_ (Supplementary Figure 1C).

### GroEL-GroES Overproducing Strains Showed Enhanced Temperature Tolerance

**FIGURE 2:**
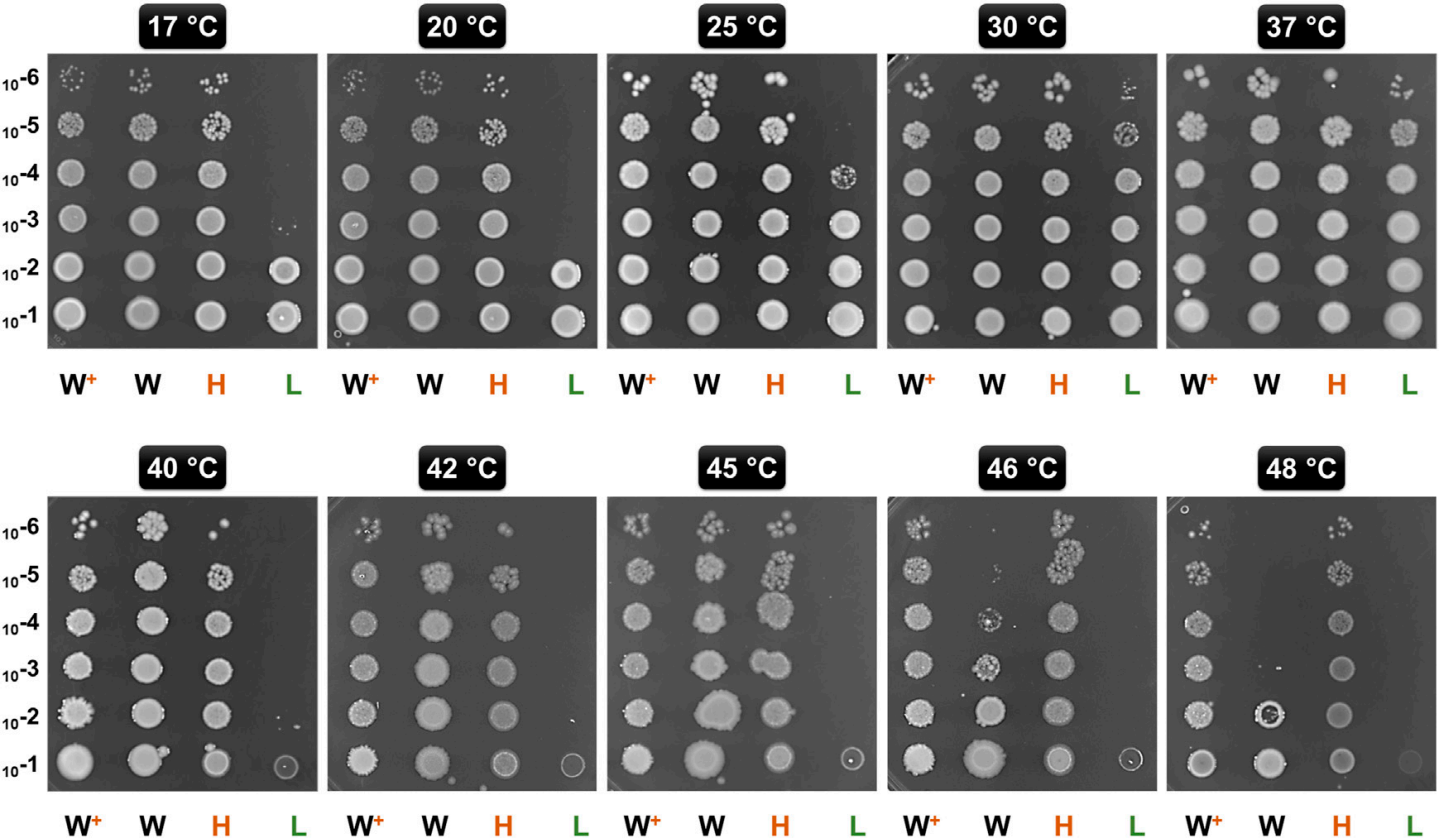
Temperature Tolerance upon Chaperonin Overproduction. Ten-fold serially diluted (10^−1^ through 10^−6^) exponentially growing cultures of GL-H_t_ (H), GL-L_t_ (L), MG16155 (W), and MG1655 with pTrc-GSL (W^+^) were spotted onto LB agar plates supplemented with lactose. These plates were incubated at the indicated temperatures.

As GroE is involved in protection against thermal stress, we analyzed the impact of different GroE levels in GL-H_t_ and GL-L_t_ on growth at temperatures ranging from 17 to 48°C (Figure 2) (Chilukoti et al., 2015). *E. coli* MG1655 and MG1655 hosting pTrc-GSL were included for comparison. As expected, GL-L_t_ cells exhibited heat and cold sensitive phenotypes and consequently showed poor growth at many temperatures, consistent with previous observations that sufficient levels of GroE are required for growth over a wide temperature range (Ferrer et al., 2003). Further, MG1655 showed much better temperature tolerance than GL-L_t_, showing the importance of the heat-shock regulation of the *P*_*groE*_ promoter. The strains harboring pTrc-GSL tolerated higher temperatures, up to 48°C, than the vector-only MG1655, where *groE* expression is temperature regulated, suggesting that higher levels of GroE enable higher temperature tolerance.

### GroEL-GroES Overproducing Strain Exhibited Fitness Advantage in Competition Culture

**FIGURE 3:**
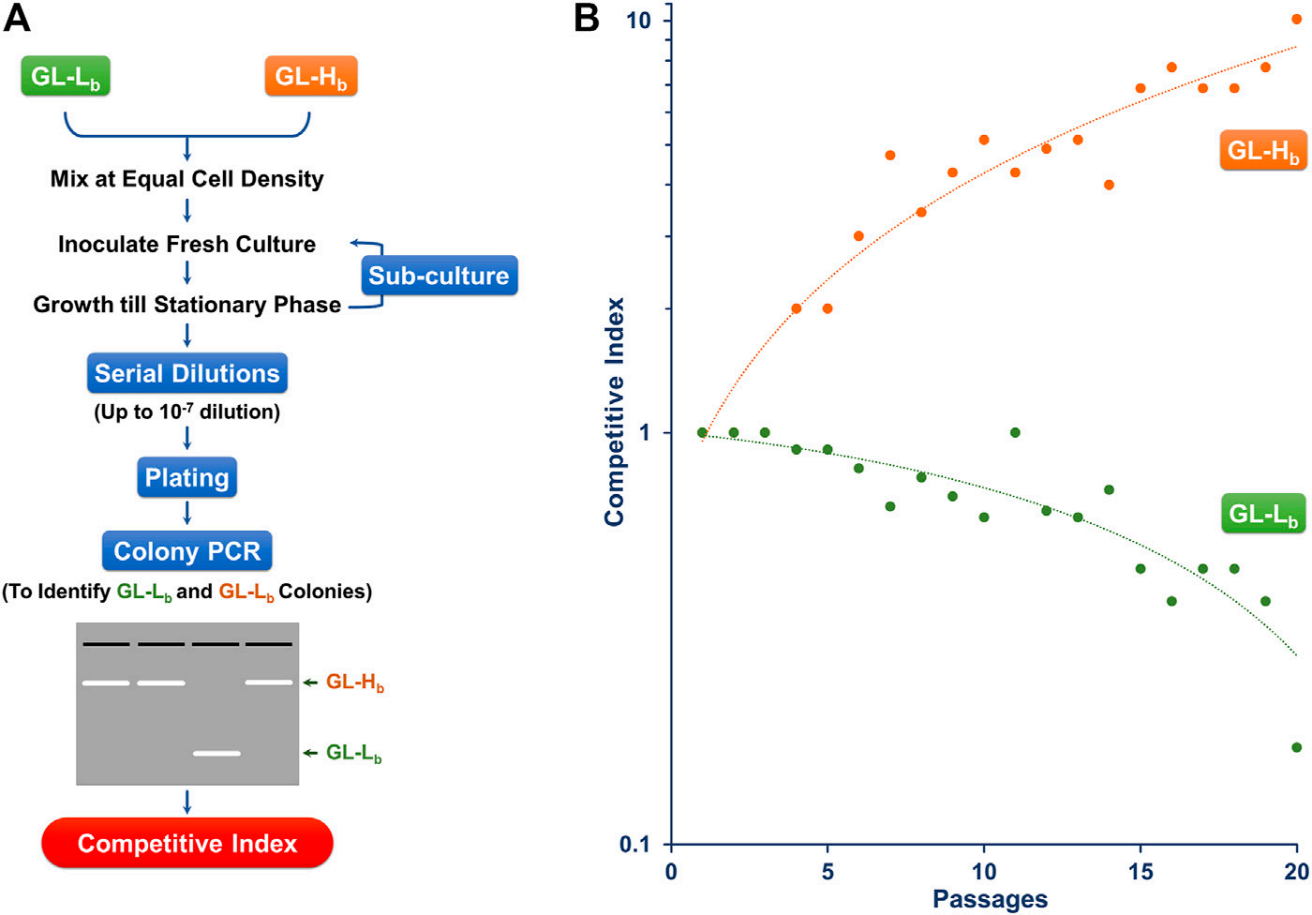
Chaperonin Depletion leads to Lower CI **(A)** Strategy for Determining the CI for GL-L_b_ and GL-H_b_ strains. Stationary phase cultures of GL-L_b_ and GL-H_b_ strains were mixed at equal cell density, grown to stationary phase and sub-cultured in fresh media for 20 continuous passages. Cells recovered at each passage were serially diluted as indicated, spread on LB agar plates and the resulting colonies were scored for their phenotype (GL-L_b_ or GL-H_b_), by colony PCR, using vector specific oligonucleotide primers **(B)** CI, as a degree of fitness, was calculated at every passage from a ratio of proportion of the cells with a particular phenotype and plotted as a function of number of passages. CI trend was similar among the three independent experiments.

Since higher levels of chaperonins led to a growth advantage, we examined whether this translated to a fitness advantage even under low stress conditions, by competing two strains with different GroE levels. Since the two strains showed similar growth profiles on the plates (Figure 2) and in independent liquid cultures at 30°C (Supplementary Figure 3), we chose this temperature for the competition culture. To do these experiments, we needed to be able to control the plasmid borne and chromosomal copies of the *groE* operon independently. Therefore, we constructed two new strains with a *P*_*BAD*_ based plasmid expression system, called GL-H_b_ (high expression) and GL-L_b_ (low expression) strains. Similar to GL-H_t_, GL-H_b_ showed several folds higher GroE induction levels (Supplementary Figure 2A) and temperature resistance (Supplementary Figure 2B). The cultures of GL-H_b_ and GL-L_b_ were competed for 20 passages (∼700 generations) and their relative fitness(s) were estimated (Figure 3A) as described in *Materials and Methods* (Monk et al., 2008; Macho et al., 2010; van Opijnen and Camilli, 2013). The high *groE* expressing GL-H_b_ outcompeted GL-L_b_ (Figure 3B), indicating that chaperonin level is an important fitness determinant.

### Proteomic Analysis Revealed Preferential Enrichment of Metabolic Enzymes in GroEL-GroES Overproducing Strains

Overproduction of a chaperonin is likely to enrich the levels of folded proteins in the cells, while unfolded or misfolded proteins tend to remain insoluble and thereby targeted to either the inclusion bodies or marked for degradation (Samuelson, 2011). Given this context, we investigated the proteomes of GL-H_b_ and GL-L_b_ cells, to identify what might account for the differences in fitness. Both strains were grown under identical conditions and their soluble proteome profiles (on 2D PAGE) were compared for relative abundance (Supplementary Figure 4; Table 1). Many of the identified proteins were known chaperonin clients belonging to either classes I and II (Kerner et al., 2005), class IV (Fujiwara et al., 2010) or the clients identified exclusively in Chapman et al. (2006), which here we have denoted as class V. However, several proteins that were identified as being differentially expressed were not known clients (Table 1), suggesting that either chaperonin overexpression can indirectly affect the folding of these non-client proteins or that the chaperonin client base is larger than currently understood. We noted that none of the obligate class III GroEL clients (Kerner et al., 2005) were relatively enriched in either strain, showing that there is sufficient chaperonin activity for folding these clients in the GL-L_b_ strain. Notably, the outer membrane proteins, OmpC and OmpF, which are involved in metabolite import and are known GroE clients (Kerner et al., 2005), were enriched in the soluble proteome of GL-L_b_. The higher level of OmpC and OmpF in the soluble fraction of GL-L_b_ suggested a lower proportion of these proteins might be reaching the outer membrane in these strains. We therefore quantified the relative levels of OmpC and OmpF in membrane fractions of both pairs of strains, and confirmed that the levels were lower in both GL-L_b_ and GL-L_t_ (Supplementary Figure 5). Further, a higher instability index (obtained from Expasy ProtParam tool), which is a reverse measure of protein stability (Guruprasad et al., 1990; Gasteiger et al., 2005) was observed for the proteins enriched in GL-H_b_ strain, suggesting that their enrichment in the chaperonin overexpressing condition may be linked to lower stability and hence a greater chaperonin requirement. The enrichment of TF in GL-L_b_ (Supplementary Figure 1A; Table 1), is consistent with previously reported interactions between TF and GroE (Kandror et al., 1995; Kandror et al., 1997) and suggests TF may be able to partially compensate for low levels of chaperonin function in GL-L_b_. Furthermore, enrichment of several metabolic enzymes in the GL-H_b_ strain, suggested a higher rate of metabolism in this strain. To evaluate this hypothesis, we collated publicly available *E. coli* proteomic data from the paxdb database (Wang et al., 2012), screened for proteins that were co-enriched with GroE across different experiments and identified 404 proteins that showed significant correlation, in expression levels, with GroE (Pearson correlation co-efficient ≥ 0.7, *p* < 0.05). Interestingly, a GO enrichment analysis of this set of proteins revealed that majority of these proteins were involved in metabolism and energy production, including multiple GO terms related to carbohydrate metabolism (Table 2).

**TABLE 1:** Properties of the differentially enriched proteins in GL-H_b_ and GL-L_b_ strains.

Strain	SwissProt entry	Protein description	MW [kDa]	pI	Unique peptides	Coverage	GroEL client class^a^	Oligomeric state	COG	SCOP fold class	Protein instability^a^	*In vivo* location	Gene	E^a^	mRNA t_1/2_ (min)
GL-H_b_	ENO_ECOLI (P0A6P9)	Enolase (EC:4.2.1.11) (2-phosphoglycerate dehydratase) (2-phospho-D-glycerate hydro-lyase)	45.5	5.32	19	51.68	One	Homodimer	G	c.1.1.1; d.54.1.1	25.64 (Stable)	Cytoplasm, cyto-skeleton, secreted, cell surface	*eno (b2779)*	1	4.7
	6PGD_ECOLI (P00350)	6-Phosphogluconate dehydrogenase, decarboxylating (EC:1.1.1.44)	51.5	5.04	16	57.48	One	Homodimer	G	a.100.1; c.2.1.6	35.98 (Stable)	Cytoplasm	*gnd (b2029)*	0	10.6
	DLDH_ECOLI (P0A9P0)	Dihydrolipoyl dehydrogenase (EC:1.8.1.4), Dihydrolipoamide dehydrogenase, E3 component of pyruvate and 2-oxoglutarate dehydrogenases complexes	50.6	5.79	18	44.51	Four	Homodimer	C	d.87.1.1; c.3.1.5	18.84 (Stable)	Cytoplasm, cell inner membrane, peripheral membrane	*lpdA (b0116)*	1	5.8
	MDH_ECOLI (P61889)	Malate dehydrogenase (EC:1.1.1.37)	32.3	5.28	19	87.5	Two	Homodimer	C	d.162.1.1; c.2.1.5	30.58 (Stable)	Cytoplasm	*mdh (b3236)*	1	10.5
	TYPH_ECOLI (P07650)	Thymidine phosphorylase (EC:2.4.2.4)	51.4	5.2	19	51.36	Four	Homodimer	F	c.27.1.1; d.41.3.1; a.46.2.1	20.63 (Stable)	Cytoplasm	*deoA (b4382)*	0	15.8
	IDH_ECOLI (P08200)	Isocitrate dehydrogenase [NADP] (EC:1.1.1.42) (Oxalosuccinate decarboxylase)	45.7	5.15	19	65.28	—	Homodimer	C	c.77.1.1	34.73 (Stable)	Cytoplasm	*icd (b1136)*	0	5.8
	ACEA_ECOLI (P0A9G6)	Isocitrate lyase (EC:4.1.3.1)	47.4	5.16	11	40.32	—	Homo-tetramer	C	c.1.12.7	36.53 (Stable)	Cytoplasm	*aceA (b4015)*	0	11.5
	DPO3B_ECOLI (P0A988)	DNA polymerase III beta subunit protein (EC:2.7.7.7)	40.5	5.45	12	40.71	—	Hetero-Oligomer	L	d.131.1.1	42.49 (Unstable)	Cytoplasm	*dnaN (b3701)*	1	2.4
	TALB_ECOLI (P0A870)	Transaldolase B (EC:2.2.1.2)	35	5.11	21	77.29	Five	Homodimer	G	c.1.10.1	31.71 (Stable)	Cytoplasm	*talB (b0008)*	0	3.4
	POTD_ECOLI (P0AFK9)	Spermidine/putrescine-binding periplasmic protein	38.8	4.86	16	50.86	—	Monomer	E	c.94.1.1	21.34 (Stable)	Periplasm	*potD (b1123)*	1	—
	RIHA_ECOLI (P41409)	Pyrimidine-specific ribonucleoside hydrolase, RihA (EC:3.2.2.-), Cytidine/uridine-specific hydrolase, ribonucleoside hydrolase 1	33.8	4.84	13	77.81	—	Tetramer	F	C.70.1.0	30.57 (Stable)	Cytoplasm	*rihA (b0651)*	0	4.4
	CH60_ECOLI (P0A6F5)	Chaperonin 60, GroEL	57	4.85	11	28.89	Five	Homo-tetradecamer	O	a.129.1.1; d.56.1.1; c.8.5.1	29.30 (Stable)	Cytoplasm	*groL (b4143)*	1	3.5
	BGAL_ECOLI (P00722)	Beta-galactosidase (EC:3.2.1.23)	116.4	5.28	51	67.68	—	Homo-tetramer	G	b.30.5.1; c.1.8.3; b.18.1.5; b.1.4.1	43.27 (Unstable)	Cytoplasm	*lacZ (b0344)*	0	10.4
GL-L_b_	TIG_ECOLI (P0A850)	Trigger factor (EC:5.2.1.8) (TF)	48.2	4.83	28	65.05	One	Homodimer and monomer	O	i.1.1.2; d.241.2.1; d.26.1.1; a.223.1.1	37.21 (Stable)	Cytoplasm	*tig (b0436)*	0	2.3
	RPOA_ECOLI (P0A7Z4)	DNA-directed RNA polymerase subunit alpha (EC:2.7.7.6) (RNAP subunit alpha), RNA polymerase subunit alpha, Transcriptase subunit alpha	36.5	4.97	15	56.53	One	Homodimer	K	d.181.1.1; i.8.1.1; a.60.3.1; d.74.3.1	41.59 (Unstable)	Cytoplasm	*rpoA (b3295)*	1	4
	PGK_ECOLI (P0A799)	Phosphoglycerate kinase (EC:2.7.2.3)	41	5.08	22	73.9	One	Monomer	G	c.86.1.1; c.1.1.1	26.37 (Stable)	Cytoplasm	*pgk (b2926)*	1	2.5
	OMPC_ECOLI (P06996)	Outer membrane protein C, outer membrane protein 1B, porin, OmpC	40.3	4.48	20	77.38	One	Homotrimer	M	f.4.3.1	12.86 (Stable)	Outer membrane	*ompC (b2215)*	0	9.7
	OMPF_ECOLI (P02931)	Outer membrane protein F, outer membrane protein 1A, outer membrane protein B, porin, OmpF	39.3	4.64	24	82.6	Two	Homotrimer	M	f.4.3.1	13.81 (Stable)	Outer membrane	*ompF (b0929)*	0	8.5
	ALF_ECOLI (P0AB71)	Fructose-bisphosphate aldolase class II (EC 4.1.2.13) (FBP aldolase), Fructose-1,6-bisphosphate aldolase	39.1	5.52	11	50.42	Two	Homodimer	G	c.1.10.2	34.82 (Stable)	Cytoplasm	*fbaA (b2925)*	1	7.2
	GLF_ECOLI (P37747)	UDP-galactopyranose mutase (EC:5.4.99.9), UDP-GALP mutase, Uridine 5-diphosphate galactopyranose mutase	43	6.61	27	79.02	Five	Homodimer	M	d.16.1.7; c.4.1.3	32.48 (Stable)	Cytoplasm	*glf (b2036)*	0	—
	SUCC_ECOLI (P0A836)	Succinyl-CoA ligase [ADP-forming] subunit beta (EC:6.2.1.5), succinyl-CoA synthetase subunit beta	41.3	5.37	22	76.8	Five	Hetero-tetramer	C	c.23.4.1; d.142.1.4	30.24 (Stable)	Cytoplasm	*sucC (b0728)*	0	6.7
	MALE_ECOLI (P0AEX9)	Maltose-binding periplasmic protein, MBP, MMBP, Maltodextrin-binding protein	43.3	5.22	13	51.77	Five	Hetero-pentamer	G	c.94.1.1	18.23 (Stable)	Periplasmic	*malE (b4034)*	0	—
	LACI_ECOLI (P03023)	Lactose operon repressor (LacI)	38.5	6.39	24	80.28	Five	Homo-tetramer	K	c.93.1.1; a.35.1.5	37.37 (Stable)	Cytoplasm	*lacI (b0345)*	0	5.7
	MANA_ECOLI (P00946)	Mannose-6-phosphate isomerase (EC:5.3.1.8), Phosphohexomutase, Phosphomannose isomerase (PMI)	42.8	5.29	16	62.92	—	Monomer	G	b.82.1.3	40.37 (Unstable)	Cytoplasm	*manA (b1613)*	0	3.6
	TREC_ECOLI (P28904)	Trehalose-6-phosphate hydrolase (EC:3.2.1.93), Alphaalpha-phosphotrehalase	63.8	5.51	31	62.61	—	—	G	c.87.1.6	33.96 (Unstable)	Cytoplasm	*treC (b4239)*	0	4.3
	AAT_ECOLI (P00509)	Aspartate aminotransferase (EC:2.6.1.1), AspAT, Transaminase A	43.5	5.54	23	61.62	Five	Homodimer	E	c.67.1.1	29.50 (Stable)	Cytoplasm	*aspC (b0928)*	0	4.3

a GroEL substrate classes 1–3 are from Kerner et al., 2005, class 4 is from Fujiwara et al., 2010, and the proteins exclusive to Chapman et al., 2006 study were denoted as class 5. Protein stability is depicted as instability index obtained from Expasy Protparam (Guruprasad et al., 1990; Gasteiger et al., 2005). Column E lists the essential (1) and non-essential (0) genes.

**TABLE 2:** Enriched Gene Ontology terms (level 3 - biological process terms), associated with the 404 proteins that were co-enriched/expressed with GroE across different experiments.

Gene ontology terms	Protein count	Fold enrichment	*p*-value	Bonferroni correction
GO:0006091: Generation of precursor metabolites and energy	76	3.810	7.95e−24	1.28e−21
GO:0044249: Cellular biosynthetic process	193	1.541	5.72e−12	9.21e−10
GO:0042180: Cellular ketone metabolic process	84	2.130	4.30e−11	6.92e−09
GO:0006082: Organic acid metabolic process	82	2.118	1.04e−10	1.68e−08
GO:0009308: Amine metabolic process	73	2.003	1.55e−08	2.50e−06
GO:0016052: Carbohydrate catabolic process	42	2.525	9.87e−08	1.59e−05
GO:0022900: Electron transport chain	26	2.992	2.28e−06	3.68e−04
GO:0006519: Cellular amino acid and derivative metabolic process	58	1.908	3.06e−06	4.92e−04
GO:0046483: Heterocycle metabolic process	45	2.052	8.50e−06	1.37e−03
GO:0006793: Phosphorus metabolic process	29	2.405	3.27e−05	5.26e−03
GO:0019538: Protein metabolic process	60	1.672	9.90e−05	1.58e−02
GO:0009059: Macromolecule biosynthetic process	114	1.355	3.68e−04	5.76e−02
GO:0006766: Vitamin metabolic process	20	2.397	7.47e−04	1.13e−01
GO:0006790: Sulphur metabolic process	18	2.540	7.89e−04	1.19e−01
GO:0016051: Carbohydrate biosynthetic process	31	1.879	1.20e−03	1.75e−01
GO:0044248: Cellular catabolic process	29	1.864	2.06e−03	2.83e−01
GO:0006461: Protein complex assembly	11	3.214	2.28e−03	3.07e−01
GO:0065003: Macromolecular complex assembly	11	3.189	2.41e−03	3.22e−01
GO:0005975: Carbohydrate metabolic process	77	1.385	2.63e−03	3.46e−01
GO:0033014: Tetrapyrrole biosynthetic process	10	3.197	4.12e−03	4.86e−01
GO:0044255: Cellular lipid metabolic process	27	1.787	5.08e−03	5.60e−01
GO:0009991: Response to extracellular stimulus	10	3.016	6.02e−03	6.22e−01
GO:0051186: Cofactor metabolic process	30	1.640	9.63e−03	7.89e−01
GO:0009057: Macromolecule catabolic process	14	2.247	9.88e−03	7.98e−01

### Flux Balance Analysis of Oxidative Phosphorylation in High- and Low-GroEL Strains.

Considering the preferential enrichment of metabolic enzymes upon GroE overproduction, we adopted an FBA approach (Orth et al., 2011; Blais et al., 2013) to assess how the differential enrichment of metabolic enzymes in the GL-L_b_ and GL-H_b_ strains would translate into altered metabolic states and cellular fitness. The FBA simulation analyses were carried out using “TransFlux” (available at: http://www.nccs.res.in/TransFlux/index.jsp), an in-house tool with a module to incorporate gene expression/proteomic profiles in the FBA framework. The proteomic profiles (Table 1) and observations from *E. coli* gene expression microarray studies, derived from the Many Microbe Microarrays database (M3D, www.m3d.mssm.edu) (Faith et al., 2008) were utilized to constrain fluxes though respective reactions, while performing two independent FBA simulations, each of which corresponded to the expression/enrichment profiles of the enzymes enriched in GL-L_b_ and GL-H_b_ strains. As expected, higher flux was observed through several pathways of carbon metabolism including glycolysis, gluconeogenesis, citric acid cycle (TCA cycle) and its anaplerotic reactions, and alternate carbon metabolism, in the simulated GL-H_b_ strain (Table 3). These pathways appear to be supported by enhanced import of glucose and glycerol (Supplementary File 2). Pathways corresponding to several glucogenic amino acids metabolism and energy generating oxidative phosphorylation were enriched in this strain. However, the pathways leading to the toxic methylglyoxal synthesis were also enriched in the GL-H_b_ strain (Table 3). We also noted that pathways leading to the metabolism of membrane lipids, pyruvic acid, pentose sugars, ubiquinone and salvage of nucleotides are enriched in the GL-L_b_ strain. Overall, FBA simulations indicated that the metabolic enzymes that were enriched in GL-H_b_ may lead to higher metabolic flux in this strain (Table 3; Supplementary Material).

**TABLE 3:** Cumulative metabolic flux through major pathways in simulated GL-L_b_ and GL-H_b_ strains as obtained through Flux Balance Analysis. Log two fold-change of fluxes of GL-H_b_ and GL-L_b_ are indicated in the Flux Ratio column.

Strain	Metabolic pathway	Metabolic flux through the pathway (mM/gm-DW/hr)^a^			
		Flux in GL-H_b_	Flux in GL-L_b_	Flux difference	Flux ratio
GL-H_b_	Glycolysis/Gluconeogenesis	289.2	59.6	229.6	2.3
	Citric acid cycle	183.9	100.1	83.7	0.9
	Oxidative phosphorylation	103.3	48.9	54.5	1.1
	Threonine and lysine metabolism	41.2	0.6	40.6	6.0
	Anaplerotic reactions	33.8	1.4	32.5	4.6
	Inorganic ion transport and metabolism	62.7	31.6	31.1	1.0
	Methylglyoxal metabolism	27.2	0.0	27.2	NA
	Transport, inner membrane	116.1	91.9	24.3	0.3
	Glutamate metabolism	25.0	3.2	21.8	3.0
	Alanine and aspartate metabolism	126.3	111.6	14.8	0.2
	Transport, outer membrane porin	28.1	17.0	11.1	0.7
	Alternate carbon metabolism	46.0	36.0	10.0	0.4
	Glycine and serine metabolism	9.8	0.8	9.0	3.6
GL-L_b_	Membrane lipid metabolism	0.4	0.4	0.0	0.0
	Cofactor and prosthetic group biosynthesis	0.0	0.0	0.0	−4.2
	Nucleotide salvage pathway	19.7	21.4	−1.7	−0.1
	Pyruvate metabolism	452.6	478.4	−25.8	−0.1
	Unassigned	0.2	30.1	−29.9	−7.0
	Pentose phosphate pathway	218.9	266.8	−47.8	−0.3

a mM/gm-DW/hr, Millimolar Metabolite per Gram Dry Weight of the cell mass per hour.

## Discussion

Over- or under-production of chaperonins in several organisms has been demonstrated to perturb rates of proteolysis (Martinez-Alonso et al., 2010), influence growth rates, and alter the expression levels of compensatory chaperones like DnaK (Lemos et al., 2007). Here we present a simple model system to study the effects of GroE overproduction (Figure 1). We demonstrate that the overexpression of GroE chaperonin results in enhanced thermal tolerance (Figure 2) and competitive advantage (Figure 3). GroEL is known to be required for growth at low (Ferrer et al., 2003) and high (Guisbert et al., 2004) temperatures. Consistent with this, the GL-L_b_ and GL-L_t_ strains exhibited both cold and heat sensitive phenotypes (Figure 2). Proteomic studies (Table 1) followed by FBA (Tables 2, 3) suggest that the acquired fitness advantage could be attributed to an enriched set of metabolic enzymes. Chaperonin depletion was observed to induce the enrichment of the compensatory chaperone, TF (Supplementary Figure 1A; Table 1), which may act as a *holdase* for the GroE client proteins (Hartl and Hayer-Hartl, 2002). Interestingly, while GroE is more abundant than TF in *E. coli* (Zou et al., 2014), TF is observed to be abundant in *mycoplasma* which lack the *groE* operon (Bang et al., 2000; Weiner et al., 2003; Musatovova et al., 2006; Lund, 2009), suggesting that higher levels of TF might be needed in such bacteria to compensate for the chaperonin deficiency. The TF - GroEL interplay, owing to their overlapping functions and client-base (Bhandari and Houry, 2015; Avellaneda et al., 2017), has been demonstrated both *in vitro* (Kandror et al., 1995) and *in vivo* in *E. coli* (Kandror et al., 1997). Therefore, it seems likely that TF enrichment in GL-L_b_ is compensating for GroE depletion and that TF may be acting on some clients as a holdase (Singhal et al., 2015). Further, the enrichment of the outer-membrane proteins OmpC and OmpF in the soluble proteome of GL-L_b_ suggests that these known GroEL client proteins failed to reach their normal final cellular destination (the outer membrane) and may have remained soluble, possibly in a TF-bound state. The reduced levels of these porins in the membranes of GL-L_b_ and GL-L_t_ strains (Supplementary Figure 5) might be responsible, in part, for the lower metabolite transport and metabolic flux in this strain (Table 3). TF was not upregulated in the wildtype strain (MG1655), despite lower GroE levels (Supplementary Figure 1), as GroE levels in this strain respond directly to levels of unfolded proteins. Furthermore, a different mode of GroE depletion resulted in the enrichment of DnaK (Calloni et al., 2012), which exhibits significant functional overlap with TF (Teter et al., 1999; Deuerling et al., 2003; Genevaux et al., 2004). The higher fitness of the GroES and GroEL over-producing strains under the conditions of our experiments is likely to be associated with fitness costs under other conditions (Figures 2, 3), otherwise it would be expected that higher expression would have evolved.

We demonstrate a direct relation between chaperonin abundance and competitive fitness. However, the evolution has not selected for intracellular chaperonin levels as high as the ones used in our experiments. The predictions from FBA simulations provide some clues that may explain why this has not occurred. Although enhanced glycolysis, TCA cycle and oxidative phosphorylation in the GL-H_b_ cells increase cellular energy currency, FBA simulations for the GL-H_b_ strain predicted an enhanced production of a toxic side product, methylglyoxal (Table 3), a very toxic three-carbon aldehyde that can inhibit *E. coli* growth at millimolar concentrations (Kayser et al., 2005; Weber et al., 2005). Therefore, evolution might have selected a balance in metabolic states between energy production and methylglyoxal toxicity, which would have, in turn, selected for an optimal level of chaperonin production. The fact that chaperonins are active ATPases provides another possible answer to this question. Overabundance of chaperonins might be linked to ATP depletion and consequent reduced growth (Sabater-Munoz et al., 2015). Thus, very high levels of chaperonin expression may have been selected against during the course of evolution. These explanations are not exhaustive, and the final level of chaperonin expression selected for is likely to result from a balance of optimizing fitness, due to multiple different factors.

Our analysis showed that GroE over-production results in several pleiotropic consequences that can enhance cellular fitness under the tested conditions. These observations need to be probed further to enhance our understanding of the precise role of the chaperone-client interactions in influencing fitness and, ultimately, evolution. A similar system could be advantageous in studying the effect of chaperonin overproduction in different microbes, especially the pathogenic bacteria with multiple chaperonins (Lund, 2009; Kumar, 2017).

## Data Availability

The original contributions presented in the study are included in the article/Supplementary Material, further inquiries can be directed to the corresponding author.
