# Supplementary material for: Chaperonin Abundance Enhances Bacterial Fitness
Source: Front Mol Biosci. 2021 Jul 26;8:669996. doi: 10.3389/fmolb.2021.669996 (PMC8350394; doi:10.3389/fmolb.2021.669996)
Supplement: Supplementary file 2 [file DataSheet1.pdf]

Chaperonin Abundance Enhances Bacterial Fitness

C. M. Santosh Kumar[a,*], Kritika Chugh[b], Anirban Dutta[c], Vishnuvardhan Mahamkali[d], Tungadri Bose[c], Sharmila S. Mande[c], and Shekhar C. Mande[e] and Peter A. Lund[a]

[a]School of Biosciences and Institute of Microbiology and Infection, University of Birmingham, B15 2TT, UK.

[b]Department of Biotechnology and Bioinformatics, University of Rajasthan, Jaipur – 302004, India.

[c]TCS Research, Tata Consultancy Services Ltd, Pune - 411013 India.

[d]Australian Institute for Bioengineering and Nanotechnology (AIBN), The University of Queensland, Brisbane - 4072, Australia

[e]Laboratory of Structural Biology, National Centre for Cell Science (NCCS), Pune - 411027, India.

* Address Correspondence to

C. M. Santosh Kumar: S.K.CM@bham.ac.uk

Running Title: Chaperonin Enhances Fitness.

Key words: Metabolic flux, Chaperonin, GroEL, Evolution, Proteomics, Metabolism

Supplementary Methods

Growth Parameters of GL-L_b and GL-H_b Strains. Overnight cultures of GL-L_b and GL-H_b strains were inoculated at equal cell number into two flasks with fresh LB supplemented with 0.2% L-arabinose and 0.2% D-lactose and were incubated with constant shaking at 30 °C. OD_{600} was measured for both the cultures at constant intervals and logarithmic values for the cell numbers at each interval were calculated. The growth parameters, such as growth rate, doubling time and growth rate constant, were calculated as per the standard calculations (Neidhardt et al., 1990). Briefly, growth rate constant was calculated as the rate of difference in the number of cells between the initial and final stages of exponential growth, using the formula, μ = (log10 N - log10 N0) X 2.303) / (t - t0). Doubling time (g) was calculated using the formula - g = (0.693/μ) X 60. Growth rate (k) was calculated by dividing μ by 0.693.

Flux Balance Analysis of the GL-L_b and GL-H_b Strains to assess metabolic changes. The *E. coli* genome-scale metabolic network iJO1366 (Orth et al., 2011) was used for the Flux Balance Analysis (FBA) simulations. Here, a simple linear relationship between the enzyme abundance (X) and the flux (F) through a corresponding reaction was assumed. TransFlux, a software tool developed in-house (available at: http://www.nccs.res.in/TransFlux/index.jsp) was used to overlay the enzyme abundance on the FBA simulations.

The iJO1366 model, the first simulated FBA model, employed standard energy source (equivalent to a glucose-supplemented minimal media) to obtain the steady state fluxes through all the reactions (Orth et al., 2011). The objective function of the FBA simulation was to maximize the biomass production, while using some 'default constraints' (lower- and upper-bounds of fluxes through each reaction) derived from the literature (Blais et al., 2013). The fluxes obtained

for each of the reactions were considered to constitute the set of 'reference fluxes' (F_R). Subsequently, for a subset of reactions, the upper-bounds (UB) and lower-bounds (LB) of the fluxes (i.e. the reaction constraints) were re-computed based on the putative expression levels of the corresponding enzyme-encoding genes (Supplementary File 2). In addition to the results from the current proteomic study, *E. coli* gene expressions, derived from the Many Microbe Microarrays Database (M3D, www.m3d.mssm.edu) (Faith et al. 2008) were screened, to gauge the possible extent of fluctuations in the expression levels of the enriched metabolic enzymes, in GL-L_b and GL-H_b strains. The ratio between the maximum and minimum expression values for specific enzymes, as obtained from the M3D, were considered as fold change (FC) and used to derive differential fluxes through corresponding reactions of the GL-L_b and GL-H_b strains. It may be noted that spots for some of the enzymes were not identified in the 2D-PAGE analysis in either GL-L_b or GL-H_b strains, although they were observed to have significant expression levels in the micro-array data. For these cases, the 'down-regulated' expression value of the enzyme was 0 (i.e., equivalent to no expression/ a deletion mutant). While re-computing the reaction constraints, it was assumed that the different expression levels of the enzyme X (say X_1 and X_2 respectively), corresponding to GL-L_b (say condition 1) and GL-H_b (say condition 2) strains, had a linear relationship with the corresponding fluxes through the reaction (F_1 and F_2, respectively). Consequently, the fold change ($FC_{1:2}$) in expression values of X_1 and X_2, would also correspond to the fold change in reaction fluxes, as shown below.

$$\frac{X_1}{X_2} = FC_{1:2} = \frac{F_1}{F_2} \ldots\ldots\ldots\ldots\ldots\ldots\ldots.(i)$$

Therefore, F_1 and F_2 may be expressed in terms of $FC_{1:2}$ as, $F_1 = F_2 * FC_{1:2} \ldots\ldots\ldots\ldots\ldots\ldots..(ii) F_2 = \frac{F_1}{FC_{1:2}} \ldots\ldots\ldots\ldots\ldots\ldots..(iii)$

It was further assumed that the reference flux (F_R) through a reaction corresponded to a condition where a given enzyme (X) was neither 'upregulated' nor 'downregulated', significantly. F_R may therefore be considered to have an intermediate value, between F_1 and F_2, and was assumed to be a simple average of F_1 and F_2, as shown below -

$$F_R = \frac{F_1 + F_2}{2} \ldots\ldots\ldots\ldots\ldots\ldots\ldots\ldots (iv)$$

From equations ii, iii and iv, the values of F_1 and F_2 may be calculated as,

$$F_1 = \frac{2 * F_R}{\left(1 + \frac{1}{FC_{1:2}}\right)} \ldots\ldots\ldots\ldots\ldots\ldots\ldots\ldots (v)$$

$$F_2 = \frac{2 * F_R}{(1 + FC_{1:2})} \ldots\ldots\ldots\ldots\ldots\ldots\ldots\ldots (vi)$$

During the conditions 1 and 2 FBA simulations, the UB and LB of the fluxes through relevant reactions were constrained based on the values of F_i (i.e., F_1 or F_2, depending on the chosen condition) and the reference flux F_R - if, $F_i > F_R$, then the LB was constrained to F_i and the UB was left unchanged; else if, $F_i < F_R$, then the UB was constrained to F_i and the LB was left unchanged.

For multi-enzyme catalyzed reactions, the effect of 'constrained bounds' were compounded using logical operators, such as 'AND' and 'OR'. While the AND operator was used to represent a scenario, wherein all the multiple enzymes were necessary to catalyze a single reaction, the OR operator represented reactions catalyzed by orthologous enzymes. Thus, a cumulative value pertaining to expression of the enzymes involved in such reactions was calculated as shown

below. This value was subsequently used for defining the LB/UB in accordance with equations described above.

$X^{ab} = min(X^a, X^b)$ … … … when enzymes 'a' AND 'b' both are essential for the reaction

$X^{ab} = max(X^a, X^b)$ … … … when either of enzymes 'a' OR 'b' is required for the reaction

Furthermore, while performing two different FBA simulations, for conditions 1 and 2, the biomass production levels of the strains were constrained into a narrow range of ± 25% of their experimentally observed values (Fig. S3).

***In silico* Identification of Proteins Co-expressed with GroE.** Publicly available proteomic data corresponding to *E. coli* was collated from the paxdb database (http://pax-db.org/) and screened for proteins potentially co-enriched with GroEL across different experiments, as below. Pearson correlation values between the expression levels of GroEL and all other protein-encoding genes were computed. Protein-encoding genes exhibiting significantly correlated expression with GroE (Pearson correlation co-efficient >= 0.7, $P < 0.05$) were selected for further analyses. Affiliations of these proteins to different COG classes were ascertained and cumulative statistics for each of the COG categories were computed (poorly characterized proteins were not considered). Moreover, Gene Ontology (GO) enrichment analysis of this set of proteins were performed with the DAVID tool (https://david.ncifcrf.gov/), to ascertain their repertoire of biological processes. GO biological process terms (level 3) satisfying a count of more than ten genes and an '*ease*' value of 0.1, were considered.

Cell Fractionation for Membrane preparation. Membrane fractions of *E. coli* GL-L_b, GL-H_b, GL- L_t and GL-H_t were isolated by standard procedures (Parham et al., 2004; Rooke et al., 2021). Briefly, the total cell

numbers for these strains were normalized to OD_{600} = 1.0, to have equal cell density in the starting samples. The bacterial cells were harvested by centrifugation at 6000 rpm for 10 min, washed twice with ice cold Buffer M (50 mM Tris.HCl (pH: 7.5), 150 mM NaCl, 1 mM EDTA + Protease inhibitor cocktail) and lysed by sonication. Unlysed cells were removed by centrifugation at 6000 rpm for 10 min. The supernatant, which contains total cellular proteins, was centrifuged at 20,000 rpm (~ 50,000 g) for 90 min in JA-25.50 Rotor in Avanti JXN-26 High-Speed Centrifuge (Beckman Coulter Inc.) to pellet the membrane fractions. The resultant pellets were resuspended in equal volume of buffer M, the membrane proteins were resolved through 12.5% SDS-PAGE and the band intensities were compared using Image J software.

Supplementary Results

Flux Balance Analysis reveals a basis for Cellular Fitness of the GroE Overproducing Strain.

The enhanced reactions in the GL-H_b and GL-L_b strains exhibited a correlation between the pathways and growth. Enhancement of the reactions of the methylglyoxal biosynthetic pathway that are known to be triggered when the cells have abundant nutrients (Kayser et al., 2005; Weber et al., 2005), further confirms the elevated energy status of the GL-H_b cells. Some of the side reactions from the amino acid metabolism, such as that catalyzed by L-threonine dehydrogenase and the follow-up reaction catalyzed by Aminoacetone:oxygen oxidoreductase (FMN), appeared to be enriched in the GL-H_b strain (Supplementary File 2). Moreover, increased excretion of L-lactic acid in combination with an enhanced glycolytic side reaction, catalyzed by L-Lactaldehyde:$NADP^+$ 1-oxidoreductase, indicated a probable increase in the intracellular methylglyoxal concentrations. Interestingly, increased excretion of the fermentation products, such as ethanol and acetic acid (Supplementary File 2), suggested the presence of an acetate

switch (Wolfe, 2005) in this strain. Although in these simulations formic acid is excreted from both the strains, excretion from GL-L_b strain was higher.

Moreover, the enrichment of L-glutamate metabolism, especially by the reaction catalyzed by glutamate dehydrogenase, the branch-point enzyme between carbon and nitrogen metabolisms catalyzing the anaplerotic reaction in the aminating direction, i. e., towards the biosynthesis of Glutamine, connected the pathways leading to the biosynthesis of the other glucogenic amino acids (Supplementary File 2). Enhanced levels of oxygen-uptake and probable increased CO_2 expulsion further supported the observed enhancement in oxidative phosphorylation reactions.

However, some of the reactions enhanced in the GL-L_b strain appear to compensate for some metabolic deficiencies in this strain. The reactions that lead to breakdown of long-chain fatty acids, such palmetoyl-CoA, stearoyl-CoA and 3-hydroxystearoyl-CoA, which are synthesized by the reactions of acetyl-CoA acyltransferase, acyl-CoA dehydrogenase and 3-hydroxyacyl-CoA dehydratase, respectively, were enriched in this strain. Moreover, the reactions leading to the salvage of nucleotides from the degradation intermediates and their feed reactions, such as ubiquinone derivative biosynthesis, pentose phosphate pathway and the reactions catalyzed by NDP kinase and NDP reductase, appeared to be enriched in this strain (Supplementary File 2). In addition, several reactions of the pyruvate metabolism that act as alternate feed reactions for TCA cycle and amino acid metabolism, were enriched. For example, the reactions catalyzed by pyruvate synthase and acetyl-CoA synthetase, that act as alternate feed reactions for L-alanine/L-asparagine biosynthesis and TCA cycle, respectively were enriched. Overall, FBA simulations (Supplementary File 2) indicated an enriched carbon, nitrogen and energy metabolism in the GL-

H_b strain, suggesting an enhancement in energy metabolism due to chaperonin overproduction and further strengthening our perception of a direct relation between chaperonins and cellular fitness.

Supplementary Discussion

The FBA revealed a correlation between chaperonin abundance and cellular metabolism. Enhanced import of metabolites and precursors into GL-H_b cells indicate that the strain can conserve energy that otherwise would be utilized in synthesizing these molecules. Moreover, FBA could predict the enhancement of nucleotide salvage pathway and its feeder pathways in GL-L_b cells (Table 3 and Supplementary File 2). Enhanced carbon and nitrogen metabolism in GL-H_b strain, accompanied with the biosynthesis of several glucogenic amino acids is predicted lead to an increase in available energy and consequent enhanced growth or stress resistance in this strain, consistent with the experimental data (Table 3 and Supplementary File 2). The GL-L_b cells apparently rely on energetically less favorable alternative pathways such as pyruvate metabolism, pentose phosphate pathway, and nucleotide salvage pathways that would feed into the major metabolic pathways in synthesizing these metabolites.

Moreover, operation of the acetate switch and the consequent increased excretion of the fermentation products, such as acetic and formic acids from the GL-H_b and GL-L_b strains (Supplementary File 2), respectively, indicate a strong correlation of metabolic and oxidative status. Accumulation and excretion of acetic acid has been demonstrated in nutrient rich and fast-growing cultures. It is triggered by excess nutrients and consequent accumulation of acetyl CoA (Wolfe, 2005). In addition to carbohydrate metabolism, enhanced flux through reaction catalyzed by acetyl CoA ligase in the GL-H_b strain (Supplementary File 2) supports the hypothesis

that GL-H_b cells might be nutrient-rich. However, increased formate accumulation has been demonstrated to increase the activity of pyruvate formatelyase (PFL) in pyruvate metabolism that converts acetyl-CoA to pyruvate (Alexeeva et al., 2000) and understandably, depletion of this central molecule of metabolism will repress several metabolic pathways, such as the TCA cycle. Notably, the said features - increased excretion of formate, increased activity of pyruvate formatelyase and depletion in TCA cycle - are apparent in the simulated GL-L_b strain (Supplementary File 2). Enhancement of the PFL reaction (Supplementary File 2) that is known to be enhanced during anaerobic growth (de Graef et al., 1999), suggests that the decreased oxidative phosphorylation flux in the GL-L_b strain leads to increased flux through mixed acid fermentation pathways. Interestingly, the differentially enriched proteins (Table 1) correlate with the acetate switch (Supplementary File 2). Maltose-binding periplasmic protein and OmpC are known to be upregulated during formate and acetate stress, respectively (Kirkpatrick et al., 2001), as observed here. Taken together, these results show how the growth and redox phenotypes of the GL-H_b and GL-L_b strains seen experimentally may arise from their respective metabolic condition as modelled in FBA from the proteomic data.

Moreover, confirming the notion that GroEL-GroES depletion results in defective membrane biogenesis (McLennan and Masters, 1998; Fujiwara and Taguchi, 2007), several reactions in membrane lipid metabolism that result in the breakdown of long chain fatty acids are enriched in the GL-L_b strain, which ultimately leads to increased pools of acetyl-CoA that can further feed to several important metabolic pathways. This also indicates that the GL-L_b strain is compromised for several metabolic pathways and thus is less robust than the GL-H_b strain. Taken

together, these studies provide direct rationales for the observed effect of chaperonins on the cellular proteome and fitness.

Supplementary Figures and Legends

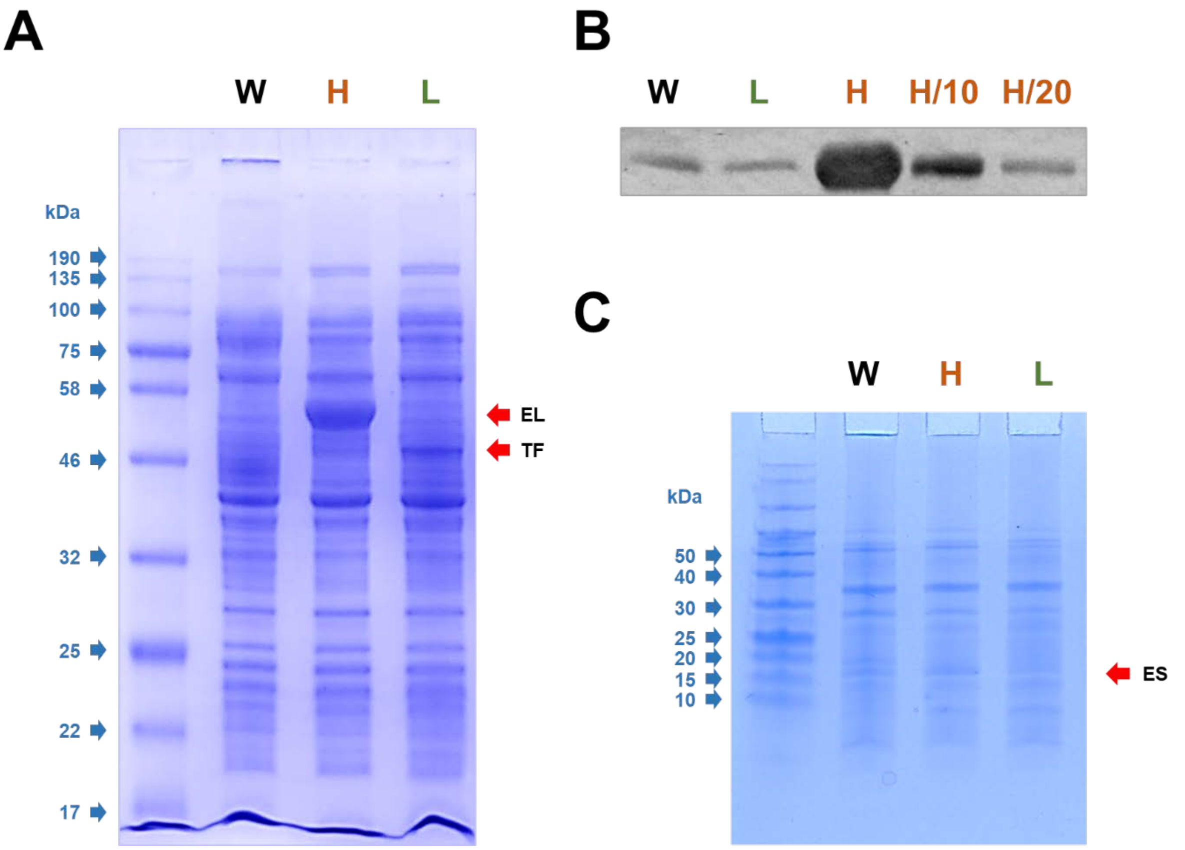

Figure S1. Validation of the GL-L_b and GL-H_b strains. A. SDS-PAGE Confirms Enhanced Levels of GroE in GL-H_t. 12.5% SDS-PAGE gel comparing the levels of GroEL (EL) in GL-H_t (H), GL-L_t (L) and the wildtype *E. coli* MG1655 (W) strains. Numbers and arrows in blue indicate molecular masses of standards in NEB Blue Protein Ladder Broad Range (#P7706). TF indicates trigger factor. **B. Immunoblotting Estimates Enhancement of GroEL in GL-Ht**. Lysates of MG1655 (W), GL-L_t (L) and GL-L_t (H) were resolved on a 12.5% SDS-PAGE and probed with GroEL specific antibody. H/10 and H/20 indicate the lanes with ten- and twenty-fold diluted GL-H_t lysates, respectively. **C.** 12 % Tricine gel comparing the levels of GroES (ES) in GL-H_t (H), GL-L_t (L) and the wildtype *E. coli* MG1655 (W) strains. GS indiacates GroES, which is known to migrate anomalously at 14.5 kDa. Numbers and arrows in blue indicate molecular masses of standards in NEB Protein Ladder (#P7703).

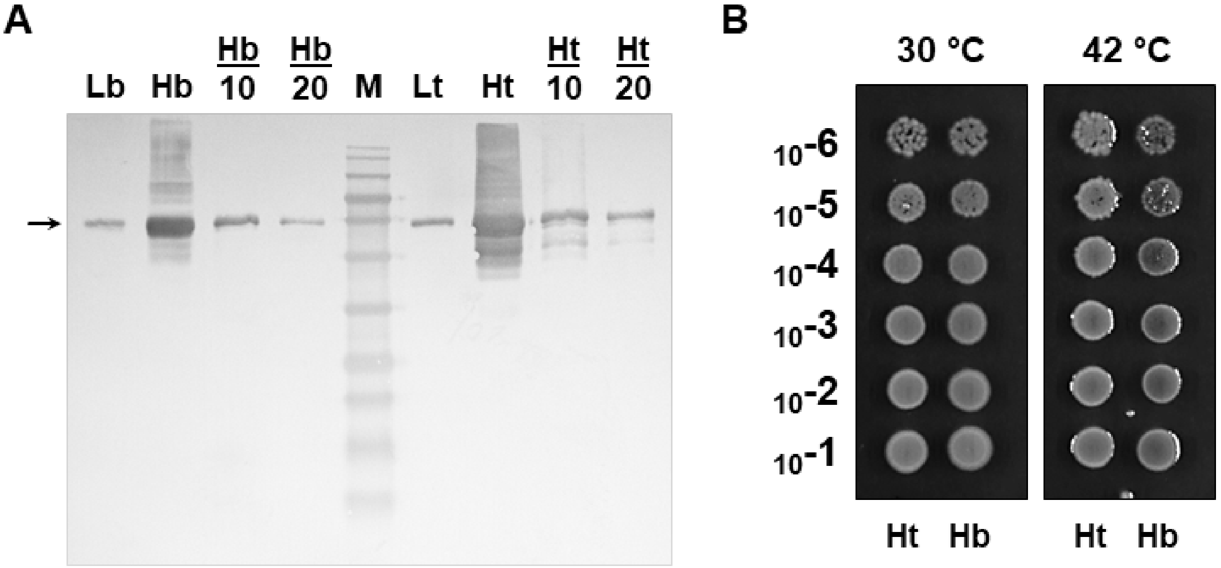

Figure S2. The pTrc99a and pBAD24 based strains show comparable characteristics. A. Enhanced GroEL production in GL-H$_t$ and GL-H$_b$ compared to their vector-only strains. Soluble lysates of GL-L$_b$ (Lb), GL-H$_b$ (Hb), GL-H$_t$ (Ht) and GL-L$_t$ (Lt) were resolved on a 12.5% SDS-PAGE and probed with GroEL specific antibody. Hb/10, Hb/20, Ht/10 and Ht/20 indicate the lanes with ten- and twenty-fold diluted lysates of GL-H$_b$ and GL-H$_t$, respectively. **B.** GL-H$_t$ and GL-H$_b$ strains exhibit similar temperature resistance. Serially diluted cultures of the GL-H$_t$ (Ht) and GL-H$_b$ (Hb) strains were spotted onto the LB agar plates supplemented with 0.2% D-lactose and 0.2% L-arabinose. The plates were incubated at the indicated temperatures.

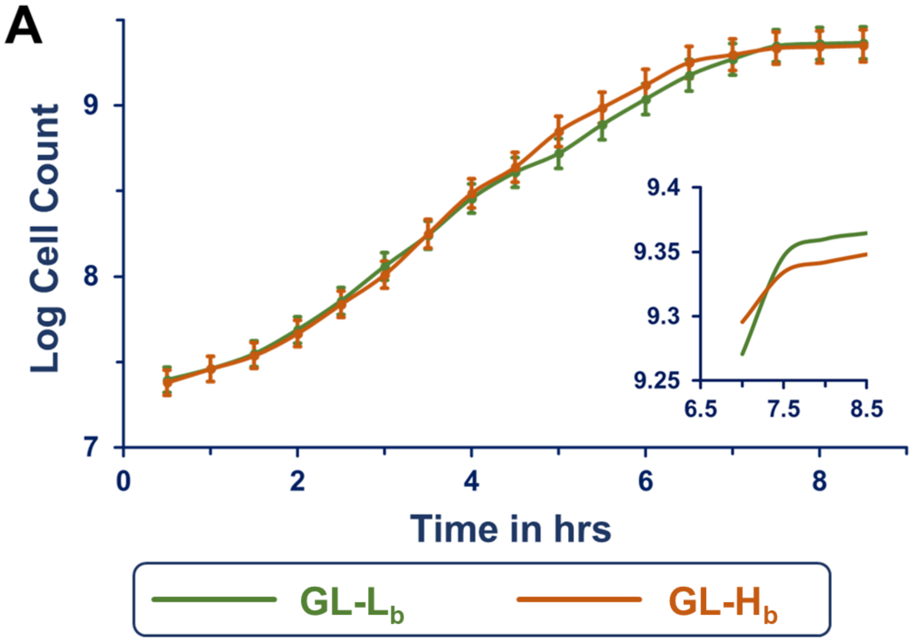

B

Strain	Growth rate constant (μ)	Growth rate (hr^{-1})	Doubling time (min)
GL-L$_b$	1.62 ± 0.12	2.33 ± 0.17	25:52 ± 1.94
GL-H$_b$	1.81 ± 0.08	2.60 ± 0.12	23:07 ± 1.08

Figure S3. GL-H$_b$ and GL-L$_b$ Strains Exhibit Comparable Growth Parameters. A. Growth curves depicting an increase in the population of the indicated strains as a function of time. The strains were cultured at 30 °C in standard LB supplemented as appropriate. The curves were fit using the least-squared method. The sub-plot with a zoomed-up region of the growth curve depicts the stationary phase of the indicated cultures. **B.** Table showing the principal growth parameters of the indicated cultures. The values are average of four independent experiments.

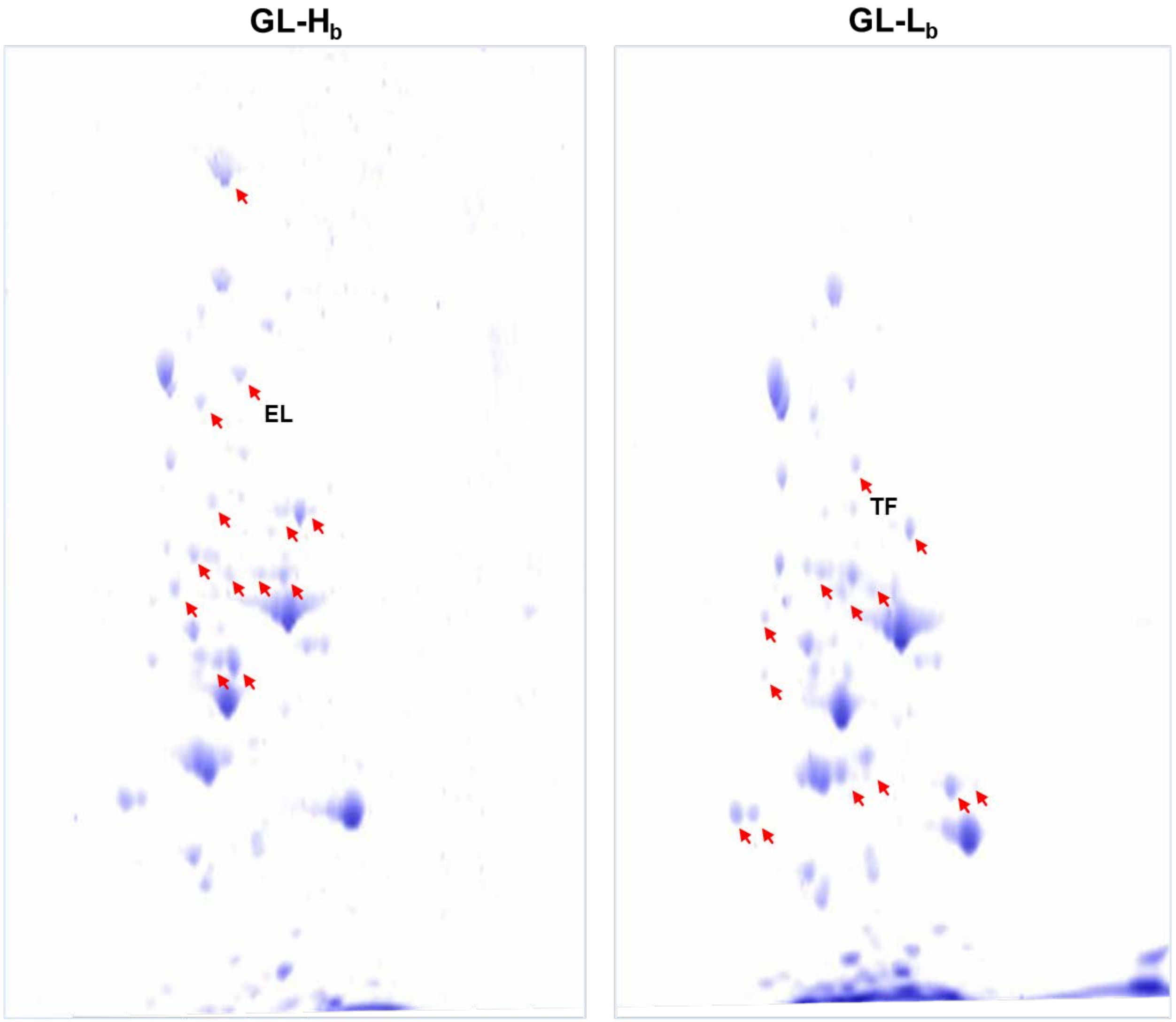

Figure S4. 2D PAGE analysis of the soluble proteomes of GL-H_b and GL-L_b Strains. Soluble lysates of **GL-H_b and GL-L_b Strains** were resolved on a 2D PAGE, with the first and second dimensions through a 3 - 10 pH gradient strip and 10% SDS-PAGE, respectively. The separated proteins were stained with Coomassie brilliant blue and intensities of the stained protein spots were compared between the two gels using densitometry. Arrows indicate differentially enriched spots. EL and TF indicate the spots identified as GroEL and Trigger Factor, respectively.

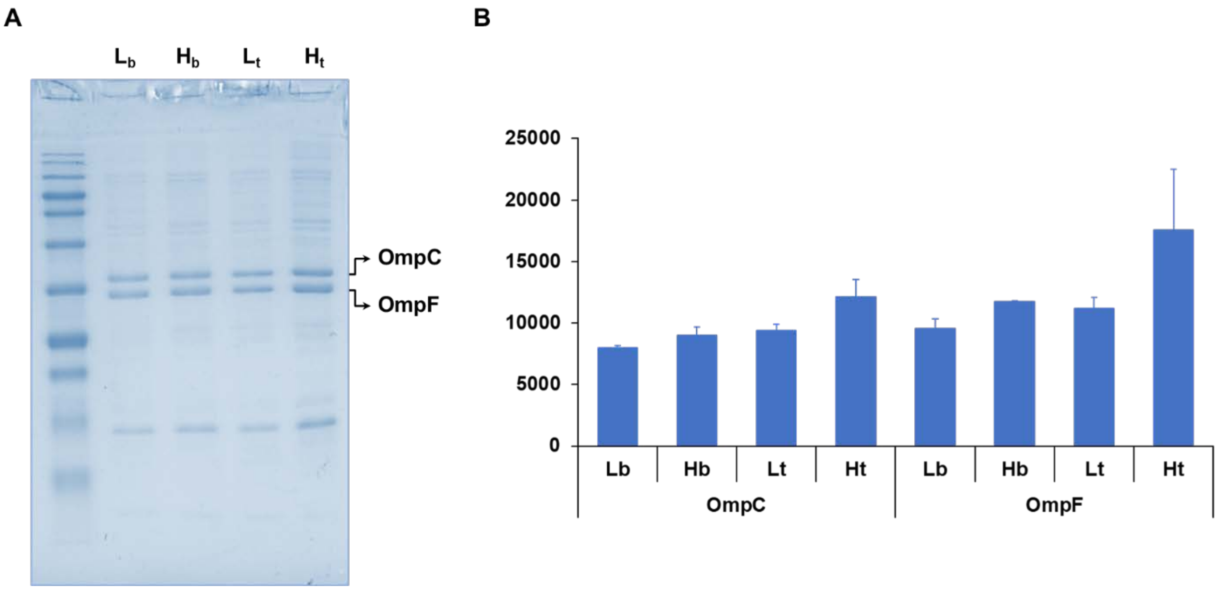

Figure S5. Cell Fractionation Revealed Differential Levels of outer membrane proteins. Cells of GL-H_b (H_b), GL-L_b (L_b), GL-H_t (H_t) and GL-L_t (L_t) strains were fractionated by high-speed centrifugation following sonication to isolate membrane fractions. These fractions were resolved on 12.5% SDS-PAGE to reveal membrane protein profiles of the indicated strains. A. Representative SDS-PAGE gel. B. Comparison of the band intensities of the indicated Outer membrane proteins.

Supplementary References.

Alexeeva, S., de Kort, B., Sawers, G., Hellingwerf, K.J., and de Mattos, M.J. (2000). Effects of limited aeration and of the ArcAB system on intermediary pyruvate catabolism in Escherichia coli. *J Bacteriol* 182(17), 4934-4940.

Blais, E.M., Chavali, A.K., and Papin, J.A. (2013). Linking genome-scale metabolic modeling and genome annotation. *Methods Mol Biol* 985, 61-83. doi: 10.1007/978-1-62703-299-5_4.

de Graef, M.R., Alexeeva, S., Snoep, J.L., and Teixeira de Mattos, M.J. (1999). The steady-state internal redox state (NADH/NAD) reflects the external redox state and is correlated with catabolic adaptation in Escherichia coli. *J Bacteriol* 181(8), 2351-2357.

Fujiwara, K., and Taguchi, H. (2007). Filamentous morphology in GroE-depleted Escherichia coli induced by impaired folding of FtsE. *J Bacteriol* 189(16), 5860-5866. doi: 10.1128/JB.00493-07.

Kayser, A., Weber, J., Hecht, V., and Rinas, U. (2005). Metabolic flux analysis of Escherichia coli in glucose-limited continuous culture. I. Growth-rate-dependent metabolic efficiency at steady state. *Microbiology* 151(Pt 3), 693-706. doi: 10.1099/mic.0.27481-0.

Kirkpatrick, C., Maurer, L.M., Oyelakin, N.E., Yoncheva, Y.N., Maurer, R., and Slonczewski, J.L. (2001). Acetate and formate stress: opposite responses in the proteome of Escherichia coli. *J Bacteriol* 183(21), 6466-6477. doi: 10.1128/JB.183.21.6466-6477.2001.

McLennan, N., and Masters, M. (1998). GroE is vital for cell-wall synthesis. *Nature* 392(6672), 139. doi: 10.1038/32317.

Neidhardt, F.C., Ingraham, J.L., and Schaechter, M. (1990). *Physiology of the Bacterial Cell: A Molecular Approach.* Sinauer Associates.

Orth, J.D., Conrad, T.M., Na, J., Lerman, J.A., Nam, H., Feist, A.M., et al. (2011). A comprehensive genome-scale reconstruction of Escherichia coli metabolism--2011. *Mol Syst Biol* 7, 535. doi: 10.1038/msb.2011.65.

Parham, N.J., Srinivasan, U., Desvaux, M., Foxman, B., Marrs, C.F., and Henderson, I.R. (2004). PicU, a second serine protease autotransporter of uropathogenic Escherichia coli. *FEMS Microbiol Lett* 230(1), 73-83. doi: 10.1016/S0378-1097(03)00862-0.

Rooke, J.L., Icke, C., Wells, T.J., Rossiter, A.E., Browning, D.F., Morris, F.C., et al. (2021). BamA and BamD Are Essential for the Secretion of Trimeric Autotransporter Adhesins. *Front Microbiol* 12, 628879. doi: 10.3389/fmicb.2021.628879.

Weber, J., Kayser, A., and Rinas, U. (2005). Metabolic flux analysis of Escherichia coli in glucose-limited continuous culture. II. Dynamic response to famine and feast, activation of the methylglyoxal pathway and oscillatory behaviour. *Microbiology* 151(Pt 3), 707-716. doi: 10.1099/mic.0.27482-0.

Wolfe, A.J. (2005). The acetate switch. *Microbiol Mol Biol Rev* 69(1), 12-50. doi: 10.1128/MMBR.69.1.12-50.2005.
